# Obligate intracellular bacteria and host cell death pathways—the matter of life and death

**DOI:** 10.1007/s00441-025-04014-9

**Published:** 2025-11-08

**Authors:** Manuela Szperlinski, Elias Schermuly, Anja Lührmann, Vera Kozjak-Pavlovic

**Affiliations:** 1 Mikrobiologisches Institut, Universitätsklinikum Erlangen, Friedrich-Alexander-Universität Erlangen-Nürnberg, Erlangen, Germany; 2 Chair of Microbiology, University of Würzburg, Biocenter, Würzburg, Germany

**Keywords:** Obligate intracellular bacteria, Cell death, Apoptosis, *Chlamydia*, *Simkania*, *Anaplasma*, *Ehrlichia*, *Orientia*, *Rickettsia*, *Coxiella*

## Abstract

To limit the damage caused by pathogenic bacteria, host organisms possess different defense systems and mechanisms for preventing infection, combating the pathogens, and creating a memory that will avert recurrent infections. Pathogens, on the other hand, have developed countermeasures to enable their replication and spreading. For intracellular pathogenic bacteria, the battleground is localized at the cellular level. Different cell types, including phagocytic, epithelial, and endothelial cells, fibroblasts, and trophoblasts, not only are equipped with diverse defense tools, but also provide different microenvironments, such as varying oxygen tension, pH levels, tonicity, and nutrient supply. The outcome of the infection depends on these conditions in conjunction with microbe-derived virulence factors and bacterial microenvironment needs. Here, we will review the current knowledge on how eukaryotic cells fight obligate intracellular bacteria and how these pathogens counteract the host cell defenses, focusing on cell death pathways. Whereas common cellular strategies for dealing with intracellular bacteria exist, there are also unique approaches adjusted to the individual properties of the pathogen.

## Introduction

**Fig. 1:**
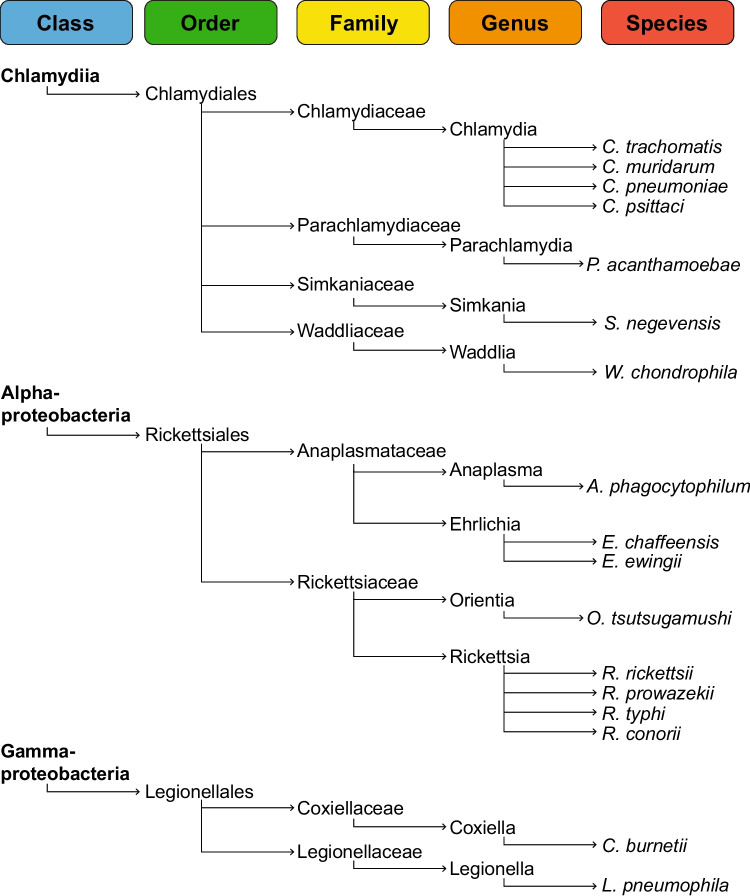
Taxonomical classification of intracellular bacteria addressed in this review. The branch lengths are not representative of genetic relatedness

Obligate intracellular bacteria depend on the ability to invade host cells and establish an intracellular niche for replication, as they lack genes allowing their replication and/or survival outside of eukaryotic cells. Thus, most of these bacteria have lost genes involved in de novo biosynthesis of metabolic intermediates, making the scavenging of nutrients from the host cells essential. While the underlying selective pressure for genome reduction is not fully understood, the advantage might be energy reduction, as the cost for biomass generation and genome replication is lowered (Mandel et al. 2024). Important members of pathogenic obligate intracellular bacteria belong to the genera *Anaplasma*, *Coxiella*, *Chlamydia*, *Ehrlichia*, *Orientia*, *Rickettsia*, and *Simkania*. Phylogenetically, these species can be divided into three orders: the Gram-negative Chlamydiales, which include *Chlamydia* and *Simkania*; the Gram-negative Alphaproteobacteria Rickettsiales, comprising the Anaplasmataceae family, with *Anaplasma* and *Ehrlichia* belonging to it, and the Rickettsiaceae family, which includes *Orientia* and *Rickettsia*; and the Gram-negative Gammaproteobacteria Legionellaes, to which *Coxiella* belongs (Fig. 1). Here, we aim to summarize our knowledge about mechanisms utilized by these obligate intracellular pathogens to interfere with the viability of their host cells. We will describe the different cell death pathways targeted by the pathogens. Furthermore, we will discuss how the individual obligate intracellular pathogens prevent and/or induce host cell death. A specific focus will be on the activity of virulence factors, mainly effector proteins of different bacterial secretion systems, which manipulate host cell death pathways.

## Programmed cell death

Apoptosis, pyroptosis, and necroptosis are the major programmed cell death pathways. While apoptosis is non-lytic and anti-inflammatory, pyroptosis and necroptosis represent inflammatory and lytic forms of cell death.

All these pathways are important for the cell to maintain cellular homeostasis as well as to fight infections. Programmed cell death pathways have to be tightly controlled as otherwise they might contribute to human diseases (Friedrich et al. 2017; Yuan and Ofengeim 2024). Failure of cell death is a hallmark of cancer. For example, many cancers are characterized by uncontrolled overexpression of anti-apoptotic Bcl-2 family members (Cory and Adams 2002). In contrast, increased cell death might lead to neurodegenerative diseases (Yuan and Ofengeim 2024), and uncontrolled necroptosis might mediate multiple sclerosis or amyotrophic lateral sclerosis (Ofengeim et al. 2015; Ito et al. 2016). During bacterial infection, unregulated cell death might lead to bacterial dissemination, and thus to the spreading of the disease within the human body (Friedrich et al. 2017).

### Apoptosis

Apoptosis does not induce inflammation and is morphologically characterized by nuclear condensation and fragmentation, blebbing of the plasma membrane, and formation of apoptotic bodies (Nagata 2018). There are two forms of apoptosis — intrinsic and extrinsic. They differ in how the apoptotic signal is sensed and transmitted.

The extrinsic apoptotic pathway is triggered by extracellular signals, which are recognized by a family of death receptors (DR). DRs, like tumor necrosis factor (TNF) receptor 1 (TNFR1), Fas, and TRAIL-R1/2, contain a death domain (DD), which allows the assembly of large complexes. The binding of ligands to Fas or TRAIL-R leads to the recruitment of FADD via DD–DD interaction. This complex is called the death-inducing signaling complex (DISC) and activates autocatalytic cleavage of procaspase-8, leading to caspase-8 activation (Dickens et al. 2012). Depending on the cell type, cleaved caspase-8 activates either caspase-3 and -7 (type I cells) or Bid (type II cells) to induce apoptosis (Jost et al. 2009).

The binding of ligands to TNFR1 might have several outcomes—it can lead to cell survival, apoptosis, or necrosis. Upon activation of TNFR1, the DD-containing protein TRADD is recruited, which in turn recruits further signaling molecules, including RIPK1, TRAF2, cIAP1/2, and caspase-8 or caspase-10. The assembled complex is ubiquitinated, which leads to the activation of the nuclear factor-κB (NF-κB) pathway and cell survival (Green and Llambi 2015). However, additional inhibition of caspase-8 triggers the necroptotic pathway (Bertheloot et al. 2021). Under caspase-8-inhibiting conditions, RIPK1 recruits RIPK3, which in turn recruits and phosphorylates MLKL. Phosphorylated MLKL can oligomerize and form pores in the plasma membrane, which ultimately leads to necroptosis (Frank and Vince 2019). Furthermore, if the ubiquitination of RIPK1 is compromised, RIPK1 and TRADD are released from the complex into the cytoplasm. This allows TRADD to interact with FADD, which in turn activates caspase-8 and, as a result, induces apoptosis (Wang et al. 2008).

The intrinsic apoptotic pathway is activated by intracellular stress, such as DNA damage, endoplasmic reticulum (ER) stress (Iurlaro and Munoz-Pinedo 2016), mitochondrial damage, or lack of nutrients (Yuan and Ofengeim 2024). This pathway is regulated by the Bcl-2 protein family, which comprises pro- and anti-apoptotic family members. The defining feature of the family members is the Bcl-2 homology (BH) domain. The anti-apoptotic members (Bcl-2, Mcl-1, Bcl-xL, Bcl-w) contain four BH domains, while the pro-apoptotic members (Noxa, Puma, Bmf, Bim, Hrk, Bik, Bad, Bid) contain only the BH3 domain (Kelekar and Thompson 1998). The pro-apoptotic members employ their BH3 domain to inhibit the anti-apoptotic Bcl-2 family members and to activate Bax and Bak. A crosstalk between extrinsic and intrinsic apoptosis pathways exists at the level of Bid. This protein can be cleaved and activated by caspase-8, leading to the recruitment of Bax and Bak to mitochondria (Yuan and Ofengeim 2024). Once activated, Bax and Bak oligomerize and form pores in the outer mitochondrial membrane, allowing the release of cytochrome *c* and activation of caspase-9, which mediates cleavage of effector caspase-3 and caspase-7. This leads to their activation and results in the cleavage of hundreds of substrates, which in turn promote apoptosis (Julien and Wells 2017).

### Pyroptosis

Pyroptosis mainly occurs in phagocytic cells and is mediated by the caspase-1 subfamily (caspase-1 and -11 in murine cells and caspase-1, caspase-4, and caspase-5 in human cells) (Yuan and Ofengeim 2024). There are two forms of pyroptosis: the canonical form and the non-canonical form.

The canonical form is activated by inflammasome receptors, which recognize danger-associated molecular patterns (DAMPs) or pathogen-associated molecular patterns (PAMPs). This leads to oligomerization and engagement of the adaptor ASC, which binds to and activates caspase-1. Activated caspase-1 cleaves not only IL1β, IL18, and gasdermin D (GSDMD), but also Bid, caspase-3, and caspase-7 (Green and Llambi 2015), indicating a crosstalk between different cell death pathways. Cleaved GSDMD oligomerizes and forms pores, allowing the release of pro-inflammatory IL1β and IL18 and ion flux, resulting in swelling and lysis of the cell.

The non-canonical inflammasome pathway is triggered by lipopolysaccharide (LPS), a component of the outer membrane of Gram-negative bacteria. Cytosolic LPS is recognized by GBP1, which initiates the assembly of caspase-11 (mouse) or caspase-4 (human) activating platform (Santos et al. 2020). Activated caspase-11 mediates cleavage of GSDMD, resulting in pore formation. Consequently, cytosolic molecules are released, which activate the canonical caspase-1-dependent inflammasome and eventually lead to cell lysis (Downs et al. 2020).

### Necroptosis

Necroptosis is a programmed cell death pathway that leads to necrosis (Degterev et al. 2008). Activation of the death receptors (DR), including TNFR1, Fas, and TRAIL-R1/2, by their ligands under apoptosis-inhibitory conditions can induce RIPK1-mediated necroptosis (Cho et al. 2009; Sun et al. 2012). Thus, activated RIPK1, under caspase-8-inhibitory conditions, binds and activates RIPK3. Activated RIPK3 mediates the phosphorylation of MLKL, which allows the oligomerization of MLKL and its interaction with phosphatidylinositol phosphates in the plasma membrane. MLKL inserts into the plasma membrane forming pores, which leads to necrosis (Li et al. 2012; Sun et al. 2012). In addition, necroptosis can result from TLR activation or in reply to metabolic stress or hypoxic conditions (He et al. 2011; Zhang et al. 2023a, b).

### Other forms of cell death

Besides apoptosis, necroptosis, and pyroptosis, other forms of cell death have been described. These include necrosis, autophagic cell death, lysosomal cell death, and ferroptosis.

Necrosis is an uncontrolled, inflammatory form of cell death. It is induced by external injury and results in the rupture of the cell membrane and the release of cellular content into the surrounding environment. In contrast to apoptosis, necrosis is energy-independent and results in inflammation and tissue damage (Elmore 2007).

Autophagic cell death is involved in the removal of aging cells and the destruction of neoplastic lesions (D'Arcy 2019). Lysosomes are organelles responsible for digesting and recycling macromolecules. They contain degradative enzymes and have an acidic lumen and heavily glycosylated membrane proteins. The rupture of the lysosomal membrane results in the release of lysosomal cathepsins, which mediate lysosomal cell death (Aits and Jaattela 2013).

Ferroptosis is driven by iron-dependent phospholipid peroxidation, which is regulated by redox homeostasis, iron handling, mitochondrial activity, and metabolism of amino acids, lipids, and sugars (Jiang et al. 2021).

In this context, it is important to mention a cellular stress response, which is activated when a large number of unfolded or misfolded proteins accumulate in the ER, the so-called unfolded protein response, or UPR. Various cellular insults, including glucose deprivation, hypoxia, oxidative stress, or infection, can lead to an imbalance in protein translation, folding, and maturation in the ER, triggering the UPR. UPR signaling aims to reduce translation, increase folding capacity, and induce misfolded protein degradation and occurs through a conserved network consisting of three branches, which involve inositol-requiring enzyme 1 (IRE1), protein kinase RNA-like ER kinase (PERK), and activating transcription factor 6 (ATF6). If the insult is prolonged or cannot be resolved, the UPR can trigger apoptosis (Hetz et al. 2020). UPR activation as a consequence of infection also leads to an inflammatory response, primarily through NF-κB and inflammasome activation (Grootjans et al. 2016), which can serve to combat bacteria but can also be subverted by pathogens to promote bacterial replication (Celli and Tsolis 2015).

During infection, intracellular pathogens often modulate one or several of the cell death pathways to ensure their survival, reproduction, and release from the host cell, which is necessary for the propagation of the infection.

## Chlamydiales

The order Chlamydiales (Fig. 1) includes obligate intracellular bacteria known for their biphasic developmental cycle, which is characterized by an infectious, environmental form (so-called elementary bodies (EB)) and a replicative, intracellular form (reticulate bodies (RB)) (Ward 2019). This order contains many species that are pathogenic to humans or animals, including well-known pathogens belonging to the genus *Chlamydia*, as well as the so-called environmental Chlamydia. These include *Protochlamydia*, *Parachlamydia*, *Simkania*, and *Waddlia*, of which many have been connected to various diseases and are considered emerging pathogens (Horn 2008).

### Chlamydia

The most prominent representative of the genus *Chlamydia* is *Chlamydia trachomatis*, a causative agent of sexually transmitted diseases (serovars D–K) (Newman et al. 2015), trachoma (serovars A–C) (Taylor et al. 2014), and lymphogranuloma venereum (serovars L1–L3) (Mabey and Peeling 2002). The life cycle of *C. trachomatis* starts with the uptake of the infective EBs, followed by the formation of a pathogen-containing vacuole, the so-called inclusion. Inside the inclusion, EBs differentiate into RBs, a replicative and metabolically active form, which re-differentiate into EBs after several rounds of division and are released from infected cells after about 36–96 h to commence the next round of infection (Ward 2019). *C. trachomatis* delivers effector proteins into the inclusion membrane (Inc proteins) or host cell cytosol using a type III secretion system (T3SS) (Betts et al. 2009). This enables the bacteria to modulate cell functions and direct nutrients to the inclusion (Rother et al. 2019), as well as protect the inclusion from destruction by the host cell defense mechanisms (Fischer and Rudel 2018).

Depending on the developmental stage, *C. trachomatis* has been shown to actively regulate host cell death. The suppression of cell death can be seen as beneficial in the early stages of infection, whereas later on, its induction is necessary for host cell lysis and the release of bacteria (Sixt 2021).

**Fig. 2:**
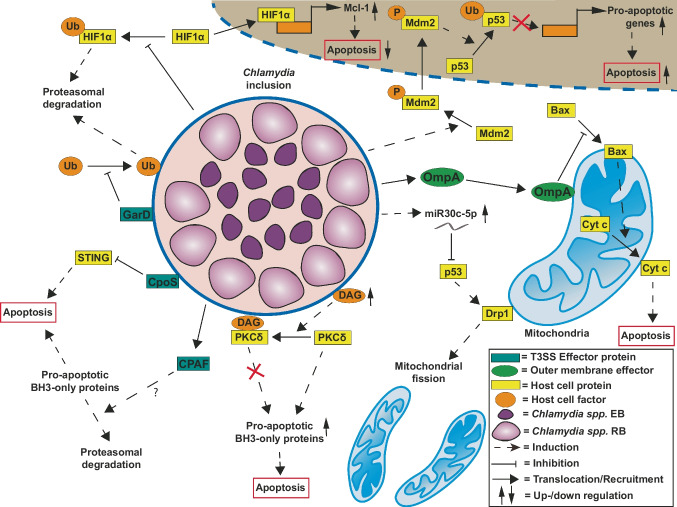
Modulation of cell death pathways by *Chlamydia *spp*. Chlamydia *spp. are known to employ several different strategies to modulate the host cell response to an infection. Several T3SS effector proteins are involved in either hampering the apoptotic response (CpoS) or maintaining the integrity of the inclusion (GarD). Other modulatory effects of *Chlamydia *spp*.* depend on structural components either of the inclusion (increased DAG) or of the bacteria themselves (OmpA). The mechanisms behind these events, such as the inhibition of pro-apoptotic genes as well as the induction of anti-apoptotic genes, are not known. The bacterial factors involved in these processes have yet to be identified. A rather unique approach for survival is the direct manipulation of host cell miRNAs to achieve a more permissive environment. The content of this figure revolves around the topics not covered in the previous review of Sixt (2021) and omits the bacterial factors involved in host cell lysis

*C. trachomatis*-infected cells are resistant to the induction of intrinsic as well as extrinsic pathways of apoptosis (Fan et al. 1998; Dean and Powers 2001; Böhme et al. 2010). This has been connected to the phosphorylation and stabilization of Myc by 3-phosphoinositide-dependent protein kinase-1 (PDPK1), leading to mitochondrial translocation of host hexokinase II (Al-Zeer et al. 2017), as well as the phosphorylation of the ubiquitin ligase Murine Double Minute 2 (Mdm2), and its interaction with p53, as shown in Fig. 2 (González et al. 2014). Interestingly, the extrinsic apoptosis induced by TNFα is blocked at the level of the internalization of TNFα/TNFR complexes, leaving the non-apoptotic TNF-signaling unaffected (Waguia Kontchou et al. 2016). However, even though *C. trachomatis-*infected cells are protected from apoptosis in early and mid-infection, they can still die by another type of cell death, which is caspase-8-dependent and resembles necrosis (Sixt et al. 2019). This shows that, though potent, the ability of *C. trachomatis* to control cell death is not limitless.

Mitochondria, as the central hubs of programmed cell death, play an important role in life cycle of *C. trachomatis*. *C. trachomatis* depends on mitochondrial ATP for development and preserves the mitochondrial network from reactive oxygen species-induced stress during infection by downregulating p53 and inhibiting Drp1-mediated mitochondrial fission. This is achieved by upregulating the microRNA miR-30c-5p (Fig. 2) (Chowdhury et al. 2017). *C. trachomatis* infection can block C95-induced apoptosis at the level of mitochondrial involvement (Fischer et al. 2004a, b) and leads to degradation of pro-apoptotic BH3-only proteins Bim/Bod, Puma, and Bad (Fischer et al. 2004a, b), as well as Bmf, Noxa, and tBid (Ying et al. 2005) upstream of the mitochondrial oligomerization of Bax/Bak. Consequently, activation of both Bax and Bak is inhibited in infected cells (Xiao et al. 2004). Staurosporine (STS)-mediated apoptosis, which relies on mitochondria and on Bax activation, is inhibited by *C. trachomatis*; however, this effect is strain-dependent, and the inhibition disappears after 48 h of infection (Jungas et al. 2004). A recent report identified several bacterial proteins that can target mitochondria and alter mitochondrial protein composition, indicating that *C. trachomatis* could modulate mitochondrial function through secreted effector proteins (Dimond et al. 2022). OmpA, a porin from the outer membrane of *C. trachomatis*, was found to associate with mitochondria and inhibit apoptosis, as evidenced by the decreased oligomerization and association of the apoptotic Bax with the outer mitochondrial membrane (Fig. 2) (Waguia Kontchou et al. 2022).

*C. trachomatis* infection inhibits apoptosis also by increasing the cellular inhibitor of apoptosis protein (cIAP)-2 (Rajalingam et al. 2006) and through upregulation and phosphatidylinositol-3-kinase (PI3K)-dependent stabilization of the anti-apoptotic Bcl-2 family member Mcl-1 (Rajalingam et al. 2008). The latter depends on the Raf/MEK/ERK pathway, which is activated upon infection together with Ca^2+^-dependent cytosolic phospholipase A2 (cPLA2) (Su et al. 2004). In addition, the HIF-1α stabilization in the early stages of infection contributes to increased Mcl-1 levels (Sharma et al. 2011). Though experiments with mouse fibroblasts lacking cIAPs or Mcl-1 contested these findings by showing that *C. trachomatis* still effectively inhibited TNFα- or STS-induced apoptosis in these cells (Ying et al. 2008), a subsequent study demonstrated that the effects of *C. trachomatis* infection were host cell-dependent (Messinger et al. 2015). Another mechanism through which *C. trachomatis* can inhibit host cell death is by scavenging pro-apoptotic effectors, such as Bad (Verbeke et al. 2006) or protein kinase C delta (PKCδ), which was found to be recruited to the vicinity of the chlamydial inclusion due to the increase of diacylglycerol in the inclusion membrane (Tse et al. 2005).

*C. trachomatis* effector proteins involved in the suppression of host cell death include the translocated actin-recruiting phosphoprotein (Tarp), which has been shown to promote apoptosis inhibition in the early stages of infection through the interaction with the SRC homology containing protein (SHC1) (Mehlitz et al. 2010). Inc protein *Chlamydia* promoter of survival (CpoS) also offers protection from cell death, since its inactivation induces rapid apoptotic and necrotic death in infected cells (Sixt et al. 2017); similar results have been reported for three more Incs, CT229, IncC, and CT383 (Weber et al. 2017). The inclusion surface harbors several other proteins that are important for chlamydial survival and control of cell death. Prevention of inclusion ubiquitination and consequent xenophagy is achieved through the action of the inclusion membrane protein gamma resistance determinant (GarD), which is shown in Fig. 2 (Walsh et al. 2022). In addition, *C. trachomatis* deubiquitinating protein ChlaDub1 acts in different ways to prevent infection-induced cell death and cell-autonomous immunity. ChlaDub1 was shown to suppress NF-κB activation induced by several pro-inflammatory stimuli and to bind the NF-κB inhibitory subunit IκBα, impairing its ubiquitination and degradation (Le Negrate et al. 2008). It is also involved in the stabilization of Mcl-1 through deubiquitination of this protein at the surface of the inclusion (Fischer et al. 2017).

Another bacterial protein that plays a role in cell death inhibition by *C. trachomatis* is chlamydial protease-like activity factor (CPAF). Its inhibition by a cell-permeable inhibitory peptide leads to the loss of inclusion integrity and caspase-1-dependent cell death (Jorgensen et al. 2011). CPAF has also been implicated in the degradation of BH3-only pro-apoptotic proteins (Fig. 2) (Pirbhai et al. 2006). The role and function of CPAF came under debate because of the difficulty in determining the real substrates of this protease (Chen et al. 2012). The generation of *C. trachomatis* mutants lacking CPAF activity should help to resolve this question (Snavely et al. 2014). Recently, CPAF has been implicated in the ability of *C. trachomatis* to prevent the activation of neutrophils and the induction of NETosis by cleaving formyl peptide receptor 2 (FPR2) (Rajeeve et al. 2018). Finally, apart from proteins, chlamydial LOS could also be involved in the inhibition of apoptosis (Wang et al. 2020a, b).

Suppression of programmed cell death has been observed during infection with other Chlamydiae as well. *C. muridarum*, *C. caviae*, and *C. psittaci* inhibit Bax and Bak activation, cytochrome *c* release, and caspase activation by STS (Zhong et al. 2006). *C. psittaci* inhibits IFN-γ-induced cell death through decreased amounts of tBid and Bim, and simultaneous upregulation of Mcl-1, which resembles *C. trachomatis* (Li et al. 2018).

*C. pneumoniae* also suppresses cell death in different cell lines (Fischer et al. 2001; Rajalingam et al. 2001; Airenne et al. 2002; Carratelli et al. 2002). Persistent *C. pneumoniae* infection makes cells resistant to TNFα- or STS-induced apoptosis, which depends on cIAPs (Paland et al. 2006). In primary human neutrophils, *C. pneumoniae* delays apoptosis (van Zandbergen et al. 2004), induces the expression of cIAP2 (Wahl et al. 2003), and activates PI3K/Akt and ERK1/2 pathways to maintain Mcl-1 expression and inhibit apoptosis (Sarkar et al. 2015). Peripheral blood monocytes exposed to *C. pneumoniae* are resistant to apoptosis induced by chemotherapeutic agents, which is dependent on the anti-inflammatory cytokine IL10 (Geng et al. 2000). In addition, *C. pneumoniae* uses host innate immune signaling through the NLRP3/ASC/caspase-1 inflammasome for intracellular growth and nutrient acquisition (Itoh et al. 2014), although caspase-1 activation and subsequent induction of IL1β production are part of the host defense against acute infection with this pathogen (He et al. 2010).

*C. muridarum*, on the other hand, has been reported to induce pyroptosis in macrophages involving both the canonical and non-canonical inflammasomes, but not RIPK3 (Finethy et al. 2015; Chen et al. 2017). However, like *C. psittaci*, *C. muridarum* can counter IFN-γ-induced programmed cell death, a mechanism important for the protection of the bacterial replicative niche (Perfettini et al. 2002a, b; Giebel et al. 2019). Another important aspect of *C. muridarum* infection is the induction of the UPR (George et al. 2017). All three UPR transducers are upregulated during *C. muridarum* infection — PERK, IRE1, and ATF6. This upregulation was linked to the increase in ATP production via substrate-level phosphorylation, autophagy induction, and apoptosis resistance, as well as the upregulation of lipid production (George et al. 2017).

In the later stages of infection, *C. trachomatis* induces cell death to promote its release from the host cell (Gibellini et al. 1998). One mechanism by which *C. trachomatis* exits infected cells is through cell lysis. It has been shown that in advanced infection, caspase-3 is activated in an manner dependent on multiplicity of infection (MOI), indicating the role of caspase signaling in this process (Matsuo et al. 2019). Host cell lysis relies on the transcriptional regulator Pgp4 encoded by the bacterial plasmid, as well as bacterial protein synthesis and the T3SS. Pgp4 is involved in the actin disposition from the inclusion membrane in late infection, which promotes bacterial release (Yang et al. 2015a, b). Another protein associated with late-stage host cell lysis is the Inc protein CTL0390, which leads to the activation of cyclic GMP-AMP synthase (cGAS) and stimulator of interferon genes (STING) (Bishop and Derré 2022). Cleaving of vimentin filaments and the nuclear envelope protein lamin-associated protein-1 (LAP1) by CPAF after disintegration of the inclusion membrane could likewise play a role in cell lysis (Snavely et al. 2014). Laser-mediated rupture of chlamydial inclusion has been shown to trigger a rapid necrotic cell death independent of caspases, Bak, Bax, or RIPK1, but depending on the presence of calcium (Kerr et al. 2017). In addition, *Chlamydia* protein associating with death domains (CADD), a redox protein toxin unique to *Chlamydia* species, has been shown to induce caspase-dependent apoptosis through interaction with TNFR1, Fas, DR4, and DR5 when expressed in mammalian cell lines (Stenner-Liewen et al. 2002; Schwarzenbacher et al. 2004).

*C. muridarum* depends on the Bax-dependent apoptosis for propagation, since the deletion of Bax diminishes the number of recovered bacteria, and Bax-/- mice clear the infection faster due to the increased inflammatory response (Perfettini et al. 2003). *C. psitacii* has also been described to induce caspase-independent cell death, which depends on Bax (Perfettini et al. 2002a, b), but the predominant way for these bacteria to exit infected host cells seems to be in a non-lytic way, in the form of *Chlamydia*-containing spheres (Scholz et al. 2024).

Unlike pathogenic representatives of the genus *Chlamydia*, environmental *Chlamydia *show a somewhat variable picture regarding the control of host cell death. The Parachlamydiaceae family members generally seem to lack potent anti-apoptotic activities in non-protozoan host cells, which limits their ability to successfully infect them (Sixt et al. 2012). A genus *Protochlamydia* member has been reported to induce apoptosis in HeLa derived Hep-2 cells, which is linked to bacterial attachment but not the formation of infection progeny in these cells and differs from other chlamydia, such as *Parachlamydia acanthamoebae* and *C. trachomatis* (Ito et al. 2012). Later, it was shown that this depends on bacterial entry, which leads to a decrease in mitochondrial membrane potential and activation of caspase-9, caspase-3, PARP cleavage, and apoptosis (Matsuo et al. 2013). Apoptosis has also been described as a defense mechanism following the infection with *P. acanthamoebae*, which has been connected to respiratory infections and atheroclerosis. Here, the inhibition of mitochondria-mediated apoptosis through the deletion of Bax/Bak or Noxa, or overexpression of Bcl-xL, supported the infection and replication of *P. acanthamoebae* (Brokatzky et al. 2020). On the contrary, *Waddlia chondrophila*, an emerging pathogen causing abortions in cattle (de Barsy and Greub 2013), has been reported not to inhibit STS-induced apoptosis, but to exhibit faster cytotoxicity than *C. trachomatis* (Dille et al. 2015).

In the next section, we will focus on one representative of environmental chlamydiae, *Simkania negevensis*.

### Simkania

*Simkania negevensis* was first described in 1993 as a cell culture contaminant of unknown origin called “Z” (Kahane et al. 1993). Subsequent phylogenetic analysis of the 16S and 23S ribosomal RNA separated *S. negevensis* into a distinct family of Simkaniaceae (Everett et al. 1999) (Fig. 1). The development of this obligate intracellular bacterium is similar to *C. trachomatis*, taking place in a compartment called the *Simkania*-containing vacuole (SnCV) and alternating between EBs and RBs, with several notable differences. Unlike *C. trachomatis*, *S. negevensis* infects a wide variety of hosts, including mammalian and arthropod cells, as well as amoeba (Kahane et al. 2001; Croxatto et al. 2014; Vouga et al. 2017a, b). Its life cycle is much longer, resulting in a development of more than 10 days without completely lysing host cells (Kahane et al. 2002). Unlike *C. trachomatis* inclusion, SnCV is a tubular structure in close contact with the ER and mitochondria (Mehlitz et al. 2014; Pilhofer et al. 2014). *S. negevensis* has been connected with respiratory tract infections (Lieberman et al. 1997; Kahane et al. 1998), lung transplant rejection (Husain et al. 2007; Jamal et al. 2015), and Crohn’s disease (Scaioli et al. 2019), though later studies have challenged this view, showing that *S. negevensis* exists rather as a commensal or opportunistic pathogen (Greenberg et al. 2003; Vouga et al. 2018).

**Fig. 3:**
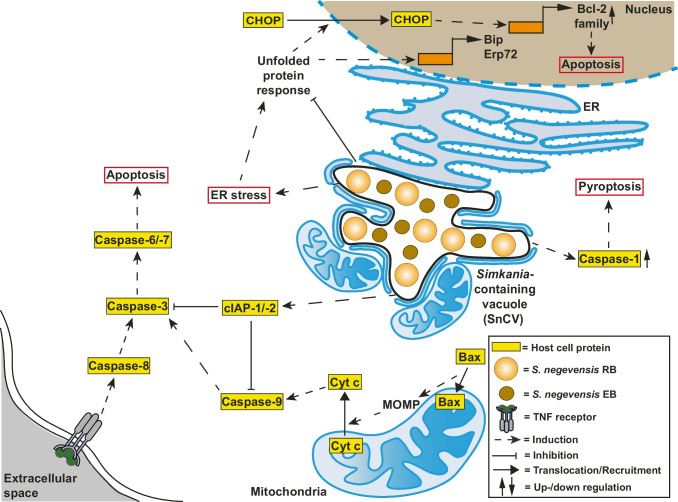
Modulation of cell death pathways by *Simkania negevensis.* Through interference with cellular stress, as well as apoptotic pathways, *S. negevensis* can create a permissive environment for intracellular replication. The possible T3SS effector proteins involved in this process are not yet identified. Nevertheless, *Simkania* interferes with the apoptotic signaling pathways and the expression of anti-apoptotic genes. Cell death might also be induced by *Simkania* through an increase in caspase-1 levels and caspase-8 activation, and increased ER stress. Importantly, the modulation of ER stress levels represents a key mechanism for apoptosis inhibition by *Simkania*

It has been reported that *S. negevensis* infection inhibits apoptosis induced by TNFα (extrinsic pathway) or STS (intrinsic pathway) at day 3 post-infection; this effect depends on the MOI and is acquired faster when more bacteria are present (Karunakaran et al. 2011). The inhibition occurs downstream of the TNF receptor activation. Caspase-3 activation and PARP cleavage are blocked, whereas caspase-8 activity is increased. The mitochondrial pathway of apoptosis generally seems to be inhibited during infection, as demonstrated by the lack of caspase-9 processing, as well as the Bax translocation or release of cytochrome *c*. In infected cells, there is no upregulation of the pro-apoptotic BH3-only proteins Bad, Bid, Puma, Bim, and Bmf, but, in contrast to *C. trachomatis*, anti-apoptotic Bcl-2 and Mcl-1 are also not upregulated (Fig. 3). When Akt signaling is inhibited, through the inhibition of PI3K, infected cells are sensitized to apoptosis; the same happens upon silencing of cIAP-1 and cIAP-2 (Fig. 3), supporting their role in the inhibition of apoptosis by *S. negevensis* (Karunakaran et al. 2011).

Whereas this study mostly focused on investigating the *S. negevensis*-mediated inhibition of apoptosis induced by TNFα, another study analyzed the role of programmed cell death in *S. negevensis* release from infected cells. The time point of infection seems to play a decisive role in changing the infection influence from cell death inhibition to cell death induction. Up to 3 days post-infection, cell death induced by TNFα, but not by STS, is efficiently blocked, as assessed by PARP cleavage (Koch et al. 2020). Starting from day 4 post-infection, cell death is actively induced by bacteria, possibly to enhance bacterial release. Interestingly, the presence of pro-apoptotic Bax, or the overexpression of anti-apoptotic Bcl-xL, does not affect *S. negevensis* release from infected cells, indicating that mitochondria-mediated cell death is not important in this process. This aspect resembles the induction of cell death upon the rupturing of chlamydial inclusion by laser, which was also not affected by the absence of Bax (Kerr et al. 2017). Taking into account the previous study (Karunakaran et al. 2011), where only caspase-8 activation was observed during infection, it is conceivable that bacteria suppress caspase-9 activation and mitochondrial permeabilization throughout infection, whereas manipulation of caspase-8 and caspase-1 signaling is required for bacterial release (Koch et al. 2020).

SnCV is a continuous membrane system, which develops in close contact with the ER; however, in contrast to *C. trachomatis,* it does not induce Golgi fragmentation (Mehlitz et al. 2014; Pilhofer et al. 2014). Although UPR is induced by *S. negevensis* in the early stage of infection, bacteria efficiently suppress it as the infection cycle progresses. *Simkania* can block both Thapsigargin- and Tunicamycin-induced ER stress, as monitored by the unchanged levels of the ER-resident chaperone immunoglobulin-binding protein BiP and a regulator of protein folding, endoplasmic reticulum–resident protein 72 (Erp72), as well as the lack of the transcription factor C/EBP homologous protein (CHOP) translocation to the nucleus, which are hallmarks of the ER stress and activation of UPR signaling (Fig. 3). However, higher quantities of Thapsigargin negatively affect the SnCV, indicating that bacterial development depends on the suppression of the ER stress response and possibly the downstream induction of cell death (Mehlitz et al. 2014).

Whereas these observations all point to *S. negevensis* actively regulating cell death signaling throughout the infection cycle, little is known about how bacteria achieve this. *S. negevensis* possesses a T3SS (Vouga et al. 2017a, b), enabling it to translocate bacterial effectors into the SnCV lumen or potentially into the host cell cytosol. *Simkania* also expresses an unusually large number of ubiquitin-modifying enzymes, with a dozen deubiquitinases (DUBs) with different ubiquitin chain specificities (Domman et al. 2014; Boll et al. 2023). Although the direct connection between this arsenal of bacterial DUBs and cell death regulation still has not been made, it is conceivable that they play an important role in the modulation of the host cell programmed death.

In conclusion, obligate intracellular bacteria of the phylum Chlamydiales that depend on their host for replication and survival often interfere with the host cell death pathways to support infection and their own development. Pathogenic members of the genus *Chlamydia* primarily inhibit cell death in the early stages of infection to ensure successful multiplication. With the progress of infection, however, bacteria induce host cell lysis to enable exit and spreading to other potential hosts. Environmental *Chlamydia* mostly fail to regulate cell death in hosts other than protozoa, which could explain their limited pathogenic potential. *S. negevensis* represents an exception, with its ability to control host cell death and stress response. Although some of the mechanisms through which this is achieved are partly conserved, specificities exist, probably linked to differences in lifestyle and host adaptation among different species.

## Rickettsiales

In the order of Rickettsiales, which consists of obligate intracellular alphaproteobacteria (Fig. 1), several important genera can be found, of which many are connected to human disease. These bacteria are known for their association with and transmission through arthropods, such as ticks, lice, fleas, and mites (McGinn and Lamason 2021). We will focus on the genera *Anaplasma*, *Ehrlichia*, *Orientia*, and *Rickettsia*, with their respective most well-studied representatives (Fig. 1).

### Anaplasma

The causative agent of human granulocytic anaplasmosis, *Anaplasma phagocytophilum*, is a tick-borne pathogen; thus, it modulates programmed cell death in both invertebrate and vertebrate hosts. However, the mechanisms seem to be adapted to the different host species (Alberdi et al. 2016). In this review, we will focus on *A. phagocytophilum*-mediated modulation of apoptosis in human cells.

**Fig. 4:**
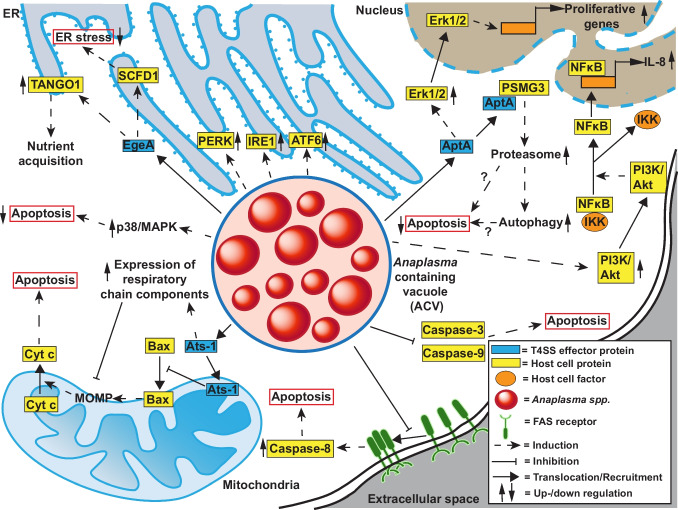
Modulation of cell death pathways by *Anaplasma* spp. The T4SS represents an important tool for *Anaplasma* spp. to manipulate host cell pathways, as several effector proteins are secreted through this mechanism and target a plethora of host cell pathways, such as the intrinsic apoptosis pathway (Ats-1) and ER stress (EgeA), as well as the induction of proliferative genes and proteasome activation (AptA). The exact mechanisms for other types of anti-apoptotic modulations, such as p38 upregulation and caspase inhibition, remain unclear

The main host cells of *A. phagocytophilum* are short-lived neutrophils. Hence, inhibition of neutrophil apoptosis is essential for *A. phagocytophilum* pathogenesis and has been observed *in vitro* and *in vivo* (Yoshiie et al. 2000; Scaife et al. 2003). Inhibition of intrinsic apoptosis of *A. phagocytophilum*-infected neutrophils is associated with increased expression of anti-apoptotic genes, activation of p38 MAPK and PI3K/Akt, inhibition of caspase-3 and caspase-9 activity, and inhibition of mitochondrial outer membrane permeabilization (MOMP) (Choi et al. 2005; Ge et al. 2005; Ge and Rikihisa 2006; Lee and Goodman 2006; Sarkar et al. 2012) (Fig. 4). Activation of the PI3K/Akt pathway also leads to NF-κB activation, resulting in IL8 secretion, which is involved in apoptosis delay (Sarkar et al. 2012). Inhibition of MOMP might be accomplished not only by inhibiting Bax translocation to mitochondria (Ge and Rikihisa 2006), but also by mitochondrial targeting of the T4SS effector protein Ats-1 (Niu et al. 2010). Ats-1-induced apoptosis inhibition is possibly mediated by hampering the redistribution of the pore-forming protein Bax and inhibiting cytochrome *c* release (Niu et al. 2010). However, Ats-1 also upregulates the expression of proteins of the respiratory chain, which might ensure the integrity of the mitochondrial membrane and the survival of the host cell (Li et al. 2022). A further T4SS effector protein, AptA, is also important for host cell survival. AptA mediates host cell survival by Erk1/2 activation (Sukumaran et al. 2011). A recent report suggests that AptA binds to the proteasome assembly chaperone 3 (PSMG3), thereby increasing the activity of the proteasome, resulting in autophagy. In cells lacking PSMG3, AptA has pro-apoptotic activity, suggesting that the binding is essential for the anti-apoptotic activity of AptA (Ma et al. 2021). However, how proteasome activation and autophagy result in apoptosis inhibition is elusive. In addition, it is unclear whether AptA-mediated Erk1/2 activation is interlinked with binding to PSMG3. Thus, further research is required to understand the molecular activity of AptA in detail. The mode of action of these effector proteins is summarized in Fig. 4.

*A. phagocytophilum* also inhibits extrinsic apoptosis. This is achieved through the inhibition of Fas clustering at the cell membrane during infection, resulting in the lack of caspase-8 activation (Ge and Rikihisa 2006).

*A. phagocytophilum* infection induces activation of all three ER stress sensors (IRE1, ATF6, and PERK). Importantly, knockdown of IRE1 accelerates apoptosis during infection, suggesting that IRE1 signaling is important for apoptosis inhibition. However, this anti-apoptotic activity does not seem to be mediated by increased XBP1 splicing (Yoshikawa et al. 2020). *A. phagocytophilum* not only activates UPR sensors, it also actively reduces ER stress and facilitates nutrient acquisition through the interaction of T4SS effector protein EgeA with the host cell proteins Transport and Golgi organization protein 1 (TANGO1) and Sec1 family domain-containing protein 1 (SCFD1) (Wang et al. 2024). This indicates that modulation of ER stress is important to maintain host cell viability (Fig. 4).

In contrast to infected neutrophils, *A. phagocytophilum-*infected human promyelocytic HL-60 cells are characterized by increased cell death (Karki and Ijdo 2011), possibly via induction of neutrophil extracellular traps (NETs) (Artigas-Jeronimo et al. 2022). This suggests that, depending on the cell type, the *A. phagocytophilum* infection might result in cell death inhibition or induction, the latter being an important defense mechanism of the host. In agreement, caspase-1 and thus pyroptosis are important for *A. phagocytophilum* control (Pedra et al. 2007). *A. phagocytophilum* infection causes phospholipase A2-mediated release of arachidonic acid, which is converted by cyclooxygenase 2 and prostaglandin E synthase 1 to prostaglandin E2 (PGE2). PGE2 signals via the EP3 receptor to activate the NLRC4 inflammasome. Thereby, caspase-1 is activated and IL1β and IL18 are secreted, which might result in pyroptosis, controlling the infection (Wang et al. 2016a, b). Importantly, tick saliva contains a molecule that impairs the formation of the NLRC4 inflammasome during infection. The tick protein sialostatin L2 binds to annexin A2 and thereby inhibits caspase-1 activation (Chen et al. 2014; Wang et al. 2016a, b). This is one example of how tick salivary proteins execute immunoregulatory activities to counteract host defense mechanisms, thereby ensuring the successful transmission of the pathogen.

### Ehrlichia

*Ehrlichia chaffeensis* is a tick-borne pathogen, which causes human monocytic ehrlichiosis (HME). HME, first reported in 1986, is a severe flu-like disease (Maeda et al. 1987). HME may lead to serious complications, affecting the function of the CNS, liver, and kidney, or cause life-threatening pneumonia. If the treatment is delayed, the mortality rate increases to 1–5 % (Paddock and Childs 2003). The main target cells of *E. chaffeensis* are mononuclear phagocytes (Paddock and Childs 2003). For host cell invasion, *E. chaffeensis* uses its virulence factor EtpE, which binds to the host cell protein DNAse X to promote entry via Neural Wiskott–Aldrich syndrome protein (N-WASP)-mediated actin polymerization (Mohan Kumar et al. 2015). Furthermore, *E. chaffeensis* utilizes caveolae-mediated endocytosis (Samanta et al. 2017). The *E. chaffeensis*-containing inclusion does not fuse with lysosomes, but instead retains features of early endosomes. Similar to *A. phagocytophilum*, *E. chaffeensis* has a biphasic lifestyle, existing in the infectious dense-core cell form (DC) and the replicative reticulate cell (RC) form. After completion of the replication cycle, *E. chaffeensis* hijacks filopodia to infect neighboring cells (Thomas et al. 2010). Major virulence factors of *E. chaffeensis* are the T4SS and the T1SS with their corresponding effector proteins (Matos et al. 2022).

**Fig. 5:**
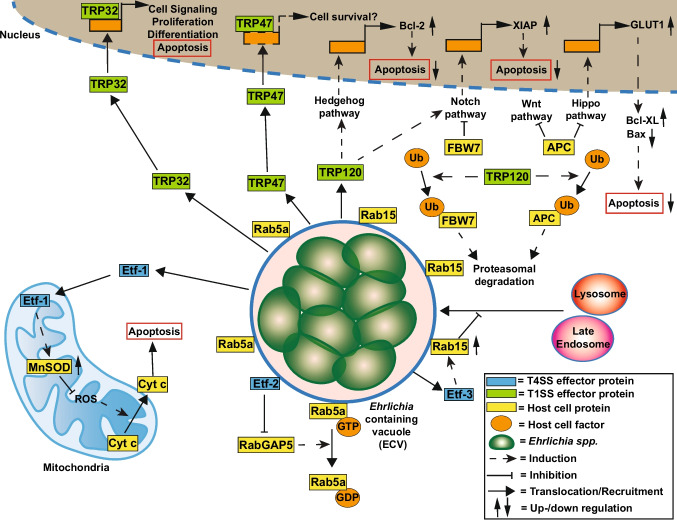
Modulation of cell death pathways by *Ehrlichia *spp*. Ehrlichia *spp. manage to avoid the degradation of their replicative compartment by the cellular machinery using different T4SS effector proteins, such as Etf-3-dependent inhibition of lysosomal recruitment and Etf-2 to prevent vacuole maturation. Interestingly, Etf-1 is not involved in these pathways, but rather in the inhibition of the intrinsic apoptosis pathway. The T1SS is also employed to secrete effector proteins, such as TRP47, TRP32, and TRP120, to ensure host cell survival

*E. chaffeensis* encodes several T1SS and T4SS effector proteins that partially modulate host cell death pathways (Fig. 5). T1SS and its effector proteins have recently been extensively reviewed (Bui et al. 2023), as well as the T4SS and its effector proteins (Rikihisa 2021). The T1SS effectors with anti-apoptotic activity include TRP32, TRP47, and TRP120. The nucleomodulin TRP32 binds to G-rich motifs and thereby modulates the expression of genes involved in not only cell signaling, proliferation, and differentiation, but also apoptosis (Farris et al. 2017). TRP47 migrates into the host cell nucleus and binds to regulatory regions of different host genes. Importantly, several targets of TRP47 overlap with those of TRP32 and TRP120, suggesting that TRP47 might also influence host cell survival (Kibler et al. 2018). However, experimental validation for this assumption has still to be provided. TRP120 modulates various signaling cascades to increase the expression of anti-apoptotic proteins. This includes the Hippo signaling pathway, which leads to upregulation of the Hippo target glucose transporter 1 (GLUT1). Higher GLUT1 levels result in increased levels of the anti-apoptotic Bcl-xL and decreased levels of the pro-apoptotic Bax (Byerly et al. 2023). Furthermore, TRP120 activates the Notch signaling cascade, which leads to increased expression of the anti-apoptotic protein XIAP (Patterson et al. 2023). In addition, TRP120 activates the Hedgehog signaling pathway, resulting in boosted Bcl-2 expression (Byerly et al. 2022). How TRP120 simultaneously interferes with the function of these different eukaryotic signaling pathways is not fully understood. However, TRP120 ligase activity, which promotes degradation of the Tumor suppressor F-box/WD repeat-containing protein 7 (FBW7), might stabilize cell survival (Wang et al. 2020a, b). Furthermore, a recent study showed that TRP120 interacts with the tumor suppressor adenomatous polyposis coli (APC), a negative regulator of Wnt and Hippo. TRP120 interaction and ubiquitination of APC result in degradation of APC and activation of Hippo and Wnt signaling (Byerly et al. 2024). These two examples suggest that TRP120 ensures host cell survival by promoting degradation of tumor suppressor proteins. The interaction of these T1SS effector proteins is shown in Fig. 5.

To date, three *E. chaffeensis* T4SS effector proteins, Etf-1, Etf-2, and Etf-3, have been identified (Rikihisa 2021). All three are required for efficient *E. chaffeensis* infection (Sharma et al. 2017; Yan et al. 2018, 2021). Etf-2 and Etf-3 are involved in establishing a replicative *E. chaffeensis*-containing vacuole (ECV). While Etf-2 interacts with Rab5 to delay maturation of the vacuole (Yan et al. 2018), Etf-3 induces Rab15 upregulation to prevent fusion of the ECV with lysosomes and to induce autophagy (Yang et al. 2024). In contrast, Etf-1 is important for inhibiting host cell apoptosis. Once translocated via the T4SS into the host cell cytoplasm, Etf-1 migrates to host mitochondria and leads to upregulation of mitochondrial manganese superoxide dismutase (MnSOD), which prevents ROS-mediated apoptosis (Liu et al. 2012). Importantly, inhibition of Etf-1 function by a specific nanobody or a macrocyclic peptide allows the control of the infection (Zhang et al. 2021; Lin et al. 2023). Hence, Etf-1 function is essential for intracellular survival and replication of *E. chaffeensis* (Fig. 5).

In case of fatal ehrlichiosis, cell death plays an important role. Apoptosis, necrosis, and inflammasome activation were observed in hepatocytes, myeloid cells, and CD4^+^T-cells (Kader et al. 2021; Ismail et al. 2022; Teymournejad et al. 2023). In addition, *Ehrlichia japonica*, which encodes homologs of several virulence factors as *E. chaffeensis*, triggers deleterious inflammasome activation (Kader et al. 2017). Importantly, inflammasome-mediated secretion of IL18 is harmful for the host, as mice deficient in the IL18 receptor are more resistant to fatal infection and have a lower bacterial burden and reduced inflammation (Ghose et al. 2011). However, caspase-1 knockout mice are highly susceptible to fatal ehrlichiosis (Yang et al. 2015a, b), suggesting that balanced inflammasome activation is crucial for the host to control the infection. This assumption is in line with the fact that Ifnar1^−/−^ mice, which are highly resistant to fatal disease, show only an attenuated activation of the inflammasome during infection (Yang et al. 2015a, b). Whether *Ehrlichia* exploits virulence factors to induce inflammasome activation to enable dissemination remains unclear.

### Orientia

*Orientia tsutsugamushi* is transmitted by larval-stage trombiculid mites and causes scrub typhus in humans, which presents as a potentially lethal febrile illness (Luce-Fedrow et al. 2018). The disease is prevalent mainly in Southeast Asia, with about one million cases annually (Bonell et al. 2017). At the site of the bite, patients develop an eschar. Further signs of the disease include fever, headache, vomiting, and lymphadenopathy (Srivastava et al. 2024). Without appropriate treatment, the disease can progress to severe complications such as multi-organ failure, renal failure, pneumonitis, acute respiratory distress, myocarditis, meningoencephalitis, septic shock, and death (Kelly et al. 2009). *O. tsutsugamushi* infects monocytes, macrophages, dendritic cells, endothelial cells, cardiomyocytes, or hepatocytes via a clathrin-mediated zipper-like mechanism (Salje 2017; Fromm et al. 2023). Once the *O. tsutsugamushi*-containing vacuole matures into a late phagosomal stage, bacteria egress into the cytosol, migrate towards the perinuclear region, and replicate within a polysaccharide-enriched microcolony (Lee et al. 2009). To exit, the pathogen uses an unusual budding mechanism, leaving the host cell encased in a portion of the plasma membrane (Kim et al. 2013). Major virulence factors of *O. tsutsugamushi* are the T4SS and the T1SS with their corresponding effector proteins (Salje 2017). *O. tsutsugamushi* also encodes for several autotransporter proteins, which, at least partially, are important for adhesion to mammalian host cells (Ha et al. 2011).

Histological studies of *Orientia*-infected tissue have shown cell death modulation by these bacteria (Panda et al. 2022). Apoptosis induction has been observed in endothelial cells, fibroblasts, neutrophils, or lymphocytes *in vitro* and *in vivo* (Kasuya et al. 1996; Kim et al. 1999; Tay et al. 2003; Liang et al. 2022). How the pathogen triggers apoptosis is mostly unknown. However, in endothelial cells, the infection results in increased expression of IL33 and its receptor. Importantly, IL33 knockout mice show reduced cellular apoptosis and less severe disease, and exogenous administration of rIL33 increases the severity of disease and decreases the expression of the anti-apoptotic Bcl-2 (Shelite et al. 2016). Hence, the alarmin IL33, as well as apoptosis induction, plays a pathogenic role in murine scrub typhus. This additionally suggests that IL33 might influence apoptosis signaling during infection.

**Fig. 6:**
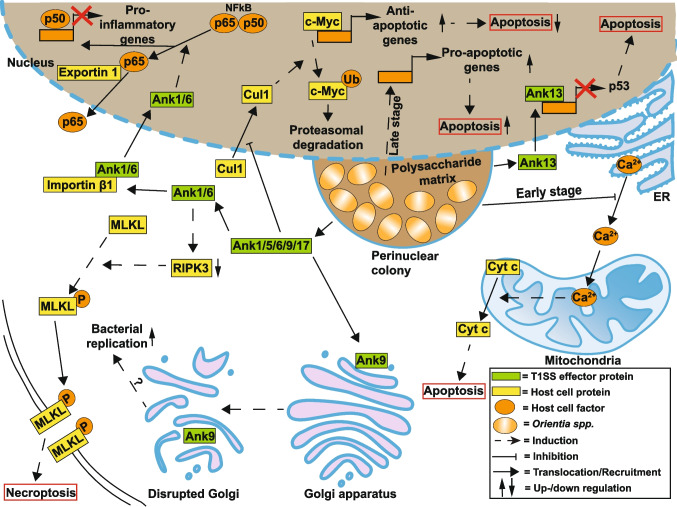
Modulation of cell death pathways by *Orientia *spp*.* Main targets of *Orientia *spp*.* are inflammatory pathways of the host cell. These pathways are modulated through several T1SS effector proteins, including Ank1 and Ank6 (manipulation of NF-κB signaling), Ank13 (downregulation of inflammatory genes), and Ank9 (Golgi-disruption). Other modulation events include the modulation of the intrinsic apoptosis pathway in the early stages of infection as well as the increased expression of pro-apoptotic genes during later infection stages

*O. tsutsugamushi* not only induces apoptosis but also inhibits host cell apoptosis. In human THP1 cells, *O. tsutsugamushi* inhibits apoptosis, possibly by preventing an increase in cytosolic calcium levels (Kim et al. 2002) (Fig. 6). This anti-apoptotic activity was observed at early stages of infection, while pro-apoptotic activity was evident after infection progression (Fromm et al. 2023). This suggests that *O. tsutsugamushi* initially prevents host cell death to ensure replication, but induces it later to allow the spread of infection. Throughout its life cycle, *O. tsutsugamushi* depends on the ability to interfere with host cell viability, which is in line with the observation that apoptosis signaling pathways are associated with single-nucleotide polymorphisms in infected patients (Kim et al. 2021).

The infection also triggers a pro-inflammatory program in human macrophages. The expression of several genes was induced in infected cells, including apoptosis-related genes and IL1β (Tantibhedhyangkul et al. 2013). IL1β secretion is mediated by inflammasome activation, which depends on ASC and caspase-1, but not on NLRP3 or NLRC4, suggesting that *O. tsutsugamushi* induces the ASC inflammasome (Fig. 6). Importantly, the infection does not induce pyroptotic cell death. On the contrary, IL1β secretion is essential for host cell control of the infection, as IL1R knockout mice are more susceptible to infection (Koo et al. 2012).

Until recently, it was unknown whether the 19 T1SS effector proteins with ankyrin repeat domains (Bui et al. 2023) are involved in modulation of host cell viability. While it was shown that Ank1 and Ank6 modulate NF-κB activity by mediating p65 nuclear export (Evans et al. 2018), whereas Ank9 perturbs the Golgi structure (Beyer et al. 2017) and Ank13 modulates host cell transcription (Adcox et al. 2021), it was uncertain whether these activities affect host cell viability. In three very recent publications, six T1SS effector proteins were identified as modulators of host cell viability (Allen et al. 2025a, b; Siff et al. 2025). Of note, Ank1, Ank5, Ank6, Ank9, and Ank17 sequester Cullin-1 in the cytoplasm, which results in elevated c-Myc nuclear levels and increased expression of anti-apoptotic genes (Allen et al. 2025a, b). Ank1 and Ank6 also reduce the expression level of RIPK3 and thereby inhibit necroptosis (Siff et al. 2025). Importantly, the nucleomodulin Ank13 executes downregulation of TP53 and inhibition of DNA damage-dependent apoptosis (Allen et al. 2025a, b). These examples demonstrate that T1SS effector proteins are essential for maintaining host cell viability during *O. tsutsugamushi* infection (Fig. 6).

In conclusion, *O. tsutsugamushi* modulates multiple host cell pathways to maintain its intracellular replicative niche during the early phase of infection. These mechanisms include blocking of *TP53* expression and thereby delaying DNA damage-dependent apoptosis (Allen et al. 2025a, b), as well as modulation of intracellular calcium levels and host cell transcription. At later stages of infection, when bacterial burden is high, *O. tsutsugamushi* induces host cell death and spread to naïve cells. Whether the induction of host cell death is an actively bacterial-driven process or host cell controlled is elusive and needs further research.

### Rickettsia

The *Rickettsia* genus (Fig. 1) is divided into several phylogenetic groups to account for its diversity. These include the spotted fever group (SFG), the typhus group (TG), the ancestral group (AG), and the transitional group (TRG), which comprise species that share both SFG and TG characteristics. Rickettsiae primarily target endothelial cells lining blood vessels, leading to vascular damage (Sahni and Rydkina 2009). They are the causative agent of a range of diseases known as rickettsioses (Hackstadt 1996; McGinn and Lamason 2021).

SFG includes bacteria typically transmitted by ticks, such as *R. rickettsii*, causing Rocky Mountain spotted fever (RMSF); *R. conorii*, the causative agent of Mediterranean spotted fever; and *R. parkeri*, which causes mild spotted fever associated with eschar. TG includes species that cause typhus fever and are spread by lice or fleas. Here, two members are of special importance: *R. prowazekii*, which causes epidemic typhus and is transmitted by body lice, and *R. typhi*, associated with rodents and spread by fleas, causing murine typhus (McGinn and Lamason 2021; Akram et al. 2025).

As obligate intracellular bacteria,* Rickettsiae* depend on the eukaryotic host cell for survival. Their life cycle starts with adherence and invasion into the host cell, mediated by the bacterial outer membrane proteins, such as OmpB (Chan et al. 2010; McGinn and Lamason 2021). After the uptake, bacteria can escape the phagocytic vacuole within minutes, which is thought to be dependent on phospholipases that break down the vacuolar membrane (Walker David et al. 1983; Silverman et al. 1992; Whitworth et al. 2005). Once in the cytosol, bacteria multiply after suppressing cellular immunity and spread from cell to cell using, in some cases, actin-based motility, similar to the mechanism applied by *Listeria monocytogenes*. To achieve this, *Rickettsiae *encode two effector proteins: RickA (Jeng et al. 2004), found in the members of SFG, AG, and TRG groups; and Sca2, found additionally in the TG representatives. In comparison to other groups, however, TG organisms display no or much shorter actin tails, leading to their accumulation in the host cell cytosol during progeny formation (Sahni and Rydkina 2009; Haglund et al. 2010; Kleba et al. 2010).

**Fig. 7:**
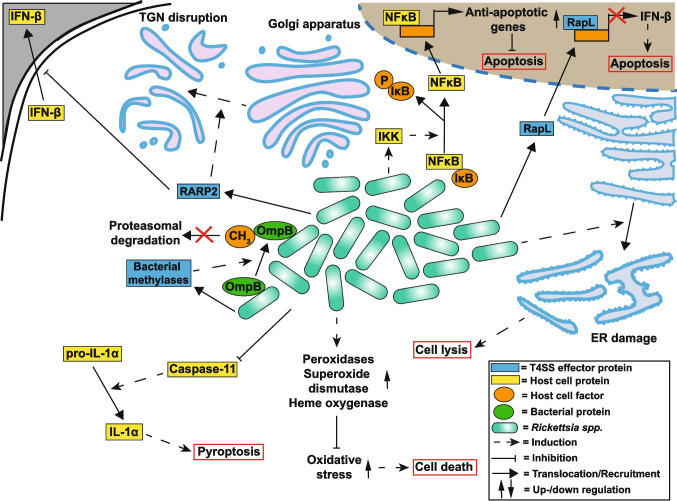
Modulation of cell death pathways by* Rickettsia *spp*.* Several effector proteins of *Rickettsia* spp. are translocated via the T4SS into the host cell and are involved in the manipulation of apoptotic signaling (RapL and RARP2) and the inhibition of bacterial degradation (bacterial methylases). Other pathways, which *Rickettsia* spp. interfere with, include NF-κB and caspase-11 signaling, as well as ROS reduction. Importantly, ER damage is also induced during the infection, highlighting a pro-apoptotic aspect of the infection

The suppression of cellular immunity by *Rickettsiae* has been shown to involve the modulation of programmed cell death and avoidance of autophagy. For example, *R. rickettsii* induces NF-κB activation through the activation of the IκB kinase (IKK) complex (Clifton et al. 2005), which is required for the inhibition of apoptosis in endothelial cells and fibroblasts (Fig. 7) (Clifton et al. 1998). The inhibition of NF-κB activation during infection induces the activation of caspase-8 and caspase-9, as well as the downstream caspase-3, but not caspase-6 and caspase-7 (Joshi et al. 2003), whereas at the same time, the pro-apoptotic Bad accumulates, and the anti-apoptotic Bcl-2 is downregulated in addition to the release of Smac and cytochrome *c* from mitochondria (Joshi et al. 2004). Caspases, however, are not activated by infection alone, implicating NF-κB activation in apoptosis suppression, which is supported by the reported resistance of *R. rickettsii*-infected endothelial cells to STS-induced apoptosis (Bechelli et al. 2009).

The avoidance of the host autophagy machinery is achieved by some *Rickettsiae*, like *R. parkeri*, through methylation of their surface proteins, which shields them from modifications such as ubiquitination (Engström et al. 2019). Two putative methyltransferases with bacterial OmpB as a target have been identified in *R. rickettsii*, implying a common mechanism for escaping autophagy (Abeykoon Amila et al. 2012). In addition, a recent report indicates a role of host glutathione (GSH) in the bacterial evasion of autophagy. GSH depletion impairs *R. parkeri* survival, prohibiting actin-based motility and spread to other cells, and diminishes the ability of bacteria to evade ubiquitination in primary macrophages. This might be related to GSH serving as a source of cysteine for bacteria (Sun et al. 2025).

Host cell lysis concludes the development of *Rickettsiae* in some cases among the members of the SFG and TG groups, but the exact bacterial factors mediating this process are unknown. Bacteria, however, encode several secretion systems, including a T4SS (Gillespie et al. 2014), which could be responsible for the delivery of effector proteins into the host cells, similar to the apoptosis modulator Ats-1 of *A. phagocytophilum* (Niu et al. 2010). *R. rickettsii* induces ER damage in endothelial cells during infection, which culminates in cell lysis 5 to 6 days post-infection (Silverman 1984). At the same time, in infected cells, intracellular peroxides accumulate, and superoxide dismutase levels (Santucci et al. 1992) as well as the levels of heme oxygenase increase (Rydkina et al. 2002), indicating that oxidative stress might contribute to cell death induced by infection.

Host cell response to infection with *Rickettsiae* includes the production of interferons (IFNs), which are immunoregulatory proteins important for immune defense. Suppression of the type-I interferon (IFN-I) response and the delay of an early cell death by a highly virulent *R. rickettsii* Sheila Smith strain are connected to more bacterial growth in human dermal microvascular endothelial cells. Infected cells die later, exhibiting activation of caspase-3/7 and cleavage of PARP and gasdermin E. Non-pathogenic *R. montanensis*, on the other hand, induces a non-apoptotic mechanism of early cell death, leading to poorer replication (Fitzsimmons et al. 2024). This study also showed that bacterial autotransporter peptidase RapL lowers IFN-β expression, whereas a rickettsial ankyrin repeat protein, RARP2 (Lehman Stephanie et al. 2018), disrupts the *trans-*Golgi network and inhibits IFN-β secretion (Fitzsimmons et al. 2024). In addition, pathogenic *Rickettsia* spp., unlike non-pathogenic, are shown to modulate inflammatory cytokine production via a caspase-11-gasdermin-dependent pathway in murine macrophages, suppressing inflammasome activation and interleukin-1 secretion (Fig. 7) (Voss et al. 2021). *R. parkeri* represents an exception. The pathogen is sensitive to IFN-I — the infection, however, leads to activation of inflammasome dependent on caspase-11, which not only results in increased host cell death but also counteracts IFN-I secretion, thereby supporting bacterial growth (Burke et al. 2020). These examples speak in favor of bacteria modulating pyroptotic cell death to manipulate inflammation and support host colonization. Finally, it is important to mention that the interaction of *Rickettsiae* with the cells of their tick vector also depends on the ability of bacteria to suppress host cell death. Not much is known about it, but a recent report has shown that *R. rickettsii* infection modulates different groups of proteins in tick cells in a time-dependent manner. This includes the increase of anti-apoptotic proteins after 48 h, the middle of the exponential phase of bacterial growth, as well as lower caspase-3 activity in infected tick cells (Martins et al. 2020).

In conclusion, suppression of cell death during infection with pathogenic *Rickettsia* plays an important role in maintaining the replicative niche of these bacteria, which show clear tropism for vascular endothelial cells and are one of the few bacterial species that reside free in the cytosol of the host cell, facing different challenges than vacuole-residing bacteria. The current lack of vaccines against major human rickettsioses emphasizes the need for more research and understanding of the interplay between host cell death pathways and intracellular *Rickettsiae*.

## Legionellales

This order from the class Gammaproteobacteria includes genera such as *Legionella* and *Coxiella*, which cause serious human infections known as Legionnaires’ disease and Q fever, respectively (Fig. 1). The most well-known species from this order is certainly *Legionella pneumophila*, but these facultative intracellular pathogens have been extensively reviewed elsewhere (Mondino et al. 2020). Here we will focus on the species with an obligate intracellular lifestyle, *Coxiella burnetii*.

### Coxiella

The first report on *C. burnetii* dates back to 1938, in which a disease of unknown origin among slaughterhouse workers in 1933 in Brisbane, Australia, was reported (Hirschmann 2019). As the etiological origin of the illness was unknown, it was termed “Q fever” after Query. Meanwhile, in 1935, an American scientist isolated an infectious agent from ticks in Nine Mile, Montana, USA. This Nine Mile agent caused symptoms similar to the Q fever agent (Davis and Cox, 1938). Three years later, a connection was made between the outbreak in Australia and the Nine Mile agent in Montana (Dyer, 1939). In 1948, the Nine Mile agent was named *Coxiella burnetii* (Hirschmann 2019). *C. burnetii* is a zoonotic pathogen, which causes coxiellosis in small ruminants and Q fever in humans (Bauer et al. 2023). In most patients, acute Q fever presents as a flu-like illness, pneumonia, or hepatitis that resolves spontaneously or after antibiotic treatment within a few weeks. However, around 2–5 % of infected individuals develop chronic Q fever, which mainly manifests as endocarditis. Treatment of chronic Q fever requires therapy with antibiotics for at least 18 months, which has limited success, with a lethality rate of 25 % (van Roeden et al. 2019). In humans, infection occurs via inhalation of contaminated dust or aerosols. The first target cells are alveolar macrophages, but *C. burnetii* can spread to other organs and other cell types. Within its host cell, *C. burnetii* generates a phagolysosomal-like *C. burnetii*-containing compartment (CCV), which fuses with autophagosomes and secretory vesicles (Campoy et al. 2011; Schulze-Luehrmann et al. 2016; Thomas et al. 2020). The bacteria multiply until the mature CCV occupies most of the host cell space (Howe et al. 2003). Egress of *C. burnetii* is ambiguous, as it happens in a non-synchronous way. Apoptosis-induction plays a role in egress, but other pathways also might be involved (Schulze-Luehrmann et al. 2024). The lipopolysaccharide (LPS) is a major determinant of virulence (Narasaki and Toman 2012). The presence of a functional T4BSS is essential for intracellular replication, indicating that T4BSS effector proteins are important virulence factors (Carey et al. 2011; Pechstein et al. 2018).

**Fig. 8:**
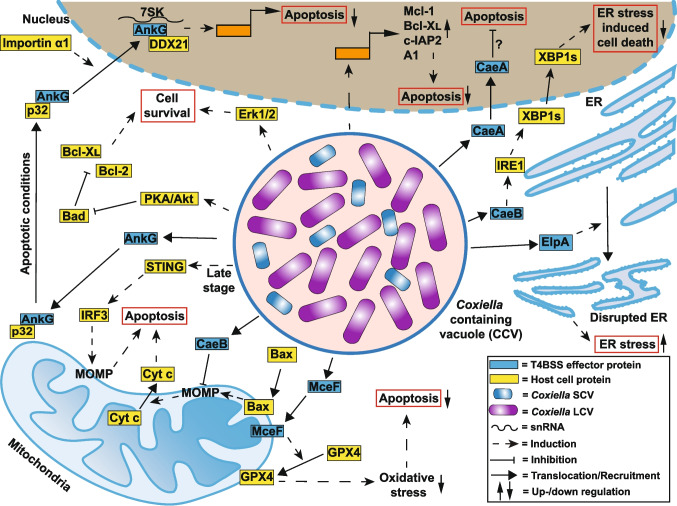
Modulation of cell death pathways by* Coxiella burnetii.* Several T4BSS effector proteins of *C. burnetii* are known to influence host cell survival pathways on many different levels. These include the inhibition of the intrinsic apoptosis pathway (CaeB) and the downregulation of pro-apoptotic genes (AnkG and CaeA). Other cell death-related pathways modulated by *Coxiella* effector proteins include the reduction of oxidative stress (MceF) and the reduction of ER stress (CaeB). Interestingly, ER stress is not only inhibited by *C. burnetii* but is also induced by ElpA. This highlights the double-edged relationship of *C. burnetii* with the manipulation of host cell apoptosis. In addition, the *C. burnetii* infection results in modulation of transcription of anti-apoptotic genes, modulation of PKA/Akt, Erk1/2, and STING pathways, which results in either inhibition or activation of host cell death

The fact that *C. burnetii* modulates apoptosis was first described in 2007 (Lührmann and Roy 2007; Voth et al. 2007). Since then, further reports demonstrate that several pathways leading to apoptosis are targeted by this pathogen (for review, see Cordsmeier et al. 2019; Osbron and Goodman 2022).* C. burnetii* requires activation of Akt, PKA, and MAPK pathways to inhibit apoptosis (Fig. 8). This might in part be accomplished by PKA and Akt-mediated phosphorylation of Bad (Voth and Heinzen 2009; Graham et al. 2013; Macdonald et al. 2014). Phosphorylation of this pro-apoptotic Bcl-2 family member prevents its binding to anti-apoptotic proteins Bcl-2 and Bcl-xL, allowing them to be active (Bui et al. 2018). In addition, several studies report that *C. burnetii* infection leads to an upregulation of anti-apoptotic genes, including Mcl-1, Bcl-xL, c-IAP-2, and A1 (Voth et al. 2007; Cherla et al. 2018). Akt might be involved in this effect (Cherla et al. 2018). These effects are illustrated in Fig. 8.

Importantly, the T4BSS is essential for *C. burnetii*-mediated apoptosis inhibition (Beare et al. 2011), pointing to the involvement of T4BSS effector proteins. Indeed, three anti-apoptotic effector proteins (AnkG, CaeA, and CaeB) have been described (Fig. 8). AnkG was the first *C. burnetii* T4BSS effector protein for which a function was assigned (Lührmann et al. 2010). Once secreted into the host cell cytosol, AnkG migrates towards the mitochondria. It binds to p32, a protein that traffics between the mitochondria and the nucleus. This allows AnkG to be transported to the host cell nucleus under apoptosis-inducing conditions (Eckart et al. 2014). To enter the host cell nucleus, AnkG requires interaction with Importin α1 (Schäfer et al. 2017, 2020). Once in the host cell nucleus, AnkG interacts with the snRNA 7SK and the RNA helicase DDX21, leading to the reprogramming of host cell transcription. Thus, the expression of anti-apoptotic genes becomes upregulated, while the expression of pro-apoptotic genes gets downregulated (Cordsmeier et al. 2022). However, AnkG does not transcriptionally modulate the abovementioned anti-apoptotic genes (Mcl-1, Bcl-xL, c-IAP-2, and A1). This suggests that AnkG is not the only bacterial factor influencing the host cell at the transcriptional level to interfere with host cell apoptosis. Indeed, *C. burnetii* encodes several nucleomodulins, which target the host cell nucleus (Bierne and Pourpre 2020; Cordsmeier et al. 2022). One of these, CaeA, inhibits intrinsic and extrinsic apoptosis (Klingenbeck et al. 2013; Bisle et al. 2016). However, how CaeA accomplishes this on the molecular level is unknown, besides the observation that its EK repetition motif is important for this process (Bisle et al. 2016). The function of these T4BSS effector proteins is shown in Fig. 8.

The CCV interacts with the ER via the mammalian lipid-binding protein ORP1L (Justis et al. 2017). This interaction seems to be important for bacterial growth and CCV expansion (Schuler et al. 2023). In general, the infection with *C. burnetii* triggers ER stress, and the T4BSS effector protein ElpA disrupts ER structures (Graham et al. 2015). However, *C. burnetii* is capable of inhibiting ER stress-induced apoptosis (Fig. 8). The T4BSS effector protein CaeB inhibits ER stress-induced cell death by upregulating the RNase activity of IRE1, resulting in increased XPB1 splicing (Friedrich et al. 2017). Importantly, *C. burnetii* infection modulates not only IRE1 but also PERK (Friedrich et al. 2017; Brann et al. 2020). PERK-mediated activation of ATF4 results in the expression of CHOP, which, upon migration to the nucleus, would regulate the expression of Bcl-2 family members, resulting in apoptotic cell death (Iurlaro and Munoz-Pinedo 2016). However, *C. burnetii* actively blocks CHOP-mediated apoptosis by inhibiting CHOP nuclear localization (Brann et al. 2020).

*C. burnetii* also controls apoptosis by preventing cytochrome *c* release from mitochondria (Lührmann and Roy 2007), suggesting that MOMP is inhibited. Indeed, CaeB interferes with MOMP (Klingenbeck et al. 2013). *C. burnetii* encodes for several T4BSS effector proteins (MceA–MceF) that target the host cell mitochondria (Fielden et al. 2017, 2021; Loterio et al. 2023). At least one of these effector proteins, MceF, protects from oxidative stress–induced cell death by recruiting the host antioxidant protein GPX4 to the mitochondria (Loterio et al. 2023). The mode of action of these previously described T4BSS effector proteins is shown in Fig. 8.

Taken together, *C. burnetii* modulates host cell transcription, signaling pathways, ER stress sensors, and mitochondrial function to prevent the execution of host cell apoptosis. The inhibition of apoptosis is of utmost importance for the pathogen during the early stages of infection, as it allows *C. burnetii* to establish a replicative niche and to produce progeny. However, *C. burnetii* not only inhibits apoptosis, but also induces it (Zhang et al. 2012). Recently, a pathway involved in *C. burnetii*-mediated apoptosis induction was identified. During the later stages of infection, STING, a cytosolic sensor for cyclic dinucleotides, activates interferon regulatory factor 3 (IRF3). Activated IRF3 interacts with Bax, which inserts into the mitochondrial membrane to induce MOMP, and thus apoptosis (Fig. 8) (Chauhan et al. 2024). Cell death after the completion of the replication cycle of *C. burnetii* results in bacterial egress, infection of bystander cells, and spreading of the infection (Schulze-Luehrmann et al. 2024). If and how effector proteins are involved in *C. burnetii*-mediated apoptosis-induction still needs to be clarified.

Gram-negative *C. burnetii* not only interferes with host cell apoptosis but might also affect pyroptosis. However, whether *C. burnetii* activates or inhibits inflammasome activation seems to depend on host cell type and species, as well as on the bacterial strain. Thus, the infection with the avirulent Nine Mile phase II (NMII) strain, but not with the virulent Nine Mile phase I (NMI) strain, triggers inflammasome activation in human alveolar macrophages (Graham et al. 2013, 2016). Importantly, in both cases, pyroptosis was not induced. Whereas NMII activates the inflammasome in human alveolar (Graham et al. 2013, 2016) and THP-1 macrophages (Palanisamy et al. 2024), as well as in murine B1a cells (Schoenlaub et al. 2016), it does not do so in murine bone marrow-derived macrophages (Cunha et al. 2015; Bradley et al. 2016; Delaney et al. 2021). Whether or not the avoidance of inflammasome activation is a bacterial-driven and/or T4BSS-dependent process is controversial (Cunha et al. 2015; Delaney et al. 2021). Whereas one report demonstrates that the T4BSS is dispensable for the observed lack of inflammasome activation during infection (Delaney et al. 2021), another report identifies the T4BSS effector protein IcaA, which inhibits caspase-11 activation and thereby prevents inflammasome activation (Cunha et al. 2015). In addition, a recent report suggests that a component of the T4BSS, icmE, activates the inflammasome (Palanisamy et al. 2024). Hence, further research is required to understand how *C. burnetii* manipulates the inflammasome pathway and thus pyroptosis.

So far, necroptosis has not been linked to *C. burnetii* infection. However, in a recent publication, the Goodman lab showed that *C. burnetii*-mediated inhibition of caspase-8 might sensitize cells to necroptosis (Osbron et al. 2024). This data suggests that the ability of *C. burnetii* to inhibit the apoptosis initiator caspase-8 might allow the cell to control the infection by lowering the threshold for necroptosis induction. However, it could also be possible that necroptosis might not allow killing of the pathogen but instead would contribute to the release of the bacteria and spreading of the infection.

## Conclusion

The multitude of mechanisms that obligate intracellular bacteria employ to interfere with host cell death pathways shows the importance of preventing host cell death during infection and indicates different levels of redundancy (Ghosh and O´Connor 2017). Pathogens might use two or more virulence factors that have the same biochemical activity or target a particular signaling pathway or cellular process. Thereby, intracellular bacteria ensure not only survival in their host cell niches but also, more importantly, the completion of their replication cycles. Of note, the described obligate intracellular pathogens are not only able to inhibit host cell death, but also to induce it. For *Anaplasma* and *Ehrlichia*, it is not clear whether cell death induction is a bacteria- or host cell-driven process. All other described pathogens are actively inducing one or multiple host cell death pathways, which might allow the generation of a microenvironment beneficial for the pathogen and might allow dissemination to other host cells or organs. Several questions still remain unclear. How do obligate intracellular pathogens switch from inhibition to induction of host cell death? How is this switch regulated? Is quorum sensing involved? Are the metabolic status or stress mediators of the host cell sensed by the pathogens, and is the metabolic state of the bacteria essential? While these questions currently lack answers, we know that the modulation of host cell death depends mainly on bacterial virulence factors, of which several are effector proteins and substrates of the bacterial secretion systems. These effector proteins are secreted into the direct environment (via T1SS) or into the cytoplasm of the host cell (via T3SS and T4SS). How the bacteria recognize which effector proteins have to be secreted at a given time point during infection is still elusive. There might be a specific order of secretion, but it might also be possible that secretion of individual effector proteins depends on the status of infection. Alternatively, it is conceivable that bacteria secrete a whole arsenal of effector proteins simultaneously, and that the outcome depends on the presence of the host cell targets. In the future, more research will be required to understand in detail how pathogens regulate communication with their host cell targets and synchronize cell death inhibition and induction with their developmental cycle. Understanding the details of this process represents not only a fascinating biological question but also a possible source of targets that can be used to combat infections with obligate intracellular bacteria.

## Data Availability

No datasets were generated or analysed during the current study.
